# Association of glycemic variability with left ventricular diastolic function in type 2 diabetes mellitus

**DOI:** 10.1186/s12933-019-0971-5

**Published:** 2019-12-05

**Authors:** Shun Yokota, Hidekazu Tanaka, Yasuhide Mochizuki, Fumitaka Soga, Kentaro Yamashita, Yusuke Tanaka, Ayu Shono, Makiko Suzuki, Keiko Sumimoto, Jun Mukai, Makiko Suto, Hiroki Takada, Kensuke Matsumoto, Yushi Hirota, Wataru Ogawa, Ken-ichi Hirata

**Affiliations:** 1 Division of Cardiovascular Medicine, Department of Internal Medicine, Kobe University Graduate School of Medicine, 7-5-2, Kusunoki-cho, Chuo-ku, Kobe, 650-0017 Japan; 2 Division of Diabetes and Endocrinology, Department of Internal Medicine, Kobe University Graduate School of Medicine, Kobe, Japan

**Keywords:** Type 2 diabetes mellitus, Glycemic variability, Diastolic function, Echocardiography

## Abstract

**Background:**

Type 2 diabetes mellitus (T2DM) is a major cause of heart failure (HF) with preserved ejection fraction (HFpEF), usually presenting as left ventricular (LV) diastolic dysfunction. Thus, LV diastolic function should be considered a crucial marker of a preclinical form of DM-related cardiac dysfunction. However, the impact of glycemic variability (GV) on LV diastolic function in such patients remains unclear.

**Methods:**

We studied 100 asymptomatic T2DM patients with preserved LV ejection fraction (LVEF) without coronary artery disease (age: 60 ± 14 years, female: 45%). GV was evaluated as standard deviation of blood glucose level using continuous glucose monitoring system for at least 72 consecutive hours. LV diastolic function was defined as mitral inflow E and mitral e’ annular velocities (E/e’), and > 14 was determined as abnormal.

**Results:**

E/e’ in patients with high GV (≥ 35.9 mg/dL) was significantly higher than that in patients with low GV (11.3 ± 3.9 vs. 9.8 ± 2.8, p = 0.03) despite similar age, gender-distribution, and hemoglobin A1c (HbA1c). Multivariate logistic regression analysis showed that GV ≥ 35.9 mg/dL (odds ratio: 3.67; 95% confidence interval: 1.02–13.22; p < 0.05) was an independently associated factor, as was age, of E/e’ > 14. In sequential logistic models for the associations of LV diastolic dysfunction, one model based on clinical variables including age, gender and hypertension was not improved by addition of HbA1c (p = 0.67) but was improved by addition of high GV (p = 0.04).

**Conclusion:**

Since HFpEF is a syndrome caused by diverse agents, reducing GV may represent a potential new therapeutic strategy for the prevention of the development of HFpEF in T2DM patients.

## Background

Type 2 diabetes mellitus (T2DM), as well as cardiovascular disease, is a major cause of heart failure (HF), both with reduced ejection fraction (HFrEF) and with preserved ejection fraction (HFpEF), [1, 2]. HF as well as T2DM is therefore one of the most worrying global public health problems [3]. Several studies have demonstrated that T2DM significantly increases the risk of recurrent HF hospitalizations and the duration of hospital stay for HF patients. The presence of T2DM is also associated with a significantly higher mortality than its absence [4]. Moreover, suboptimal glycemic control and impaired insulin sensitivity characteristic of T2DM have also been found to be directly correlated with an increased risk of developing HF [5, 6]. T2DM is also a major cause of HFpEF, while LV diastolic function was found to be strongly associated with HFpEF [7, 8]. It is well known that patients with HF face a risk of similar magnitude regardless of their EF status because there is currently no effective pharmacological therapy for patients with HFpEF. Interest has therefore been growing in a reliable therapy for HFpEF or in improvement of LV diastolic function for T2DM patients with preserved LVEF since these may lead to more effective prevention of the development of HFpEF in such patients. On the other hand, glycemic variability (GV) has specific clinical implications, as well as a different significance than that of classical markers such as hemoglobin A1c (HbA1c) [9]. A continuous glucose monitoring system (CGM) is an emerging technology that can continuously measure glucose levels, thereby enabling evaluation of GV. It has been widely known that GV is strongly associated with the progression of coronary artery disease in T2DM patients [10]. However, it remains uncertain if GV represent a potential new therapeutic strategy for the prevention of the development of HFpEF in asymptomatic T2DM patients with preserved LVEF. The objective of this study was, therefore, to investigate the impact of GV on the LV diastolic function of such patients.

## Methods

A total of 100 DM patients were retrospectively enrolled in this study. They had been admitted to Kobe University Hospital between July 2013 and September 2015 and undergone both echocardiography and CGM. Preliminary exclusion criteria for this study were: (1) history of coronary artery disease; (2) LVEF < 50%; (3) previous history of open-heart surgery or congenital heart disease; (4) severe renal dysfunction defined as glomerular filtration rate < 30 mL/min/1.73 m^2^; (5) uncontrolled hypertension > 180/100 mmHg; (6) more than moderate valvular heart disease; and (7) atrial fibrillation. All enrolled patients underwent an exercise stress screening test such as a treadmill exercise or stress myocardial perfusion scintigraphy during their hospitalization, and patients with an ischemic response were excluded. The diagnosis of T2DM was based on the World Health Organization criteria [11]. This study was approved by the local ethics committee of our institution (No. 180332).

### Echocardiographic examination

All echocardiographic data were obtained by means of a commercially available echocardiographic system (Vivid E9; GE-Vingmed, Horten, Norway) within 2 weeks after admission. Digital routine grayscale two-dimensional cine loops from three consecutive heart beats were obtained at end-expiratory apnea from standard parasternal and apical views. Sector width was optimized to allow for complete myocardia visualization while maximizing the frame rate. Standard echocardiographic measurements were obtained according to the current guidelines of the American Society of Echocardiography (ASE)/European Association of Cardiovascular Imaging (EACVI) [12]. Specifically, the early diastolic (E) and atrial wave (A) velocities and the E-wave deceleration time were measured by means of pulsed wave Doppler recording from the apical four-chamber view. Spectral pulsed-wave Doppler-derived early diastolic velocity (e′) was obtained by averaging the septal and lateral mitral annulus, and the E/e′ ratio was then calculated to obtain an estimate of LV filling pressure. In particular, E/e’ > 14 was evaluated as LV diastolic dysfunction as recommended by the ASE/EACVI [13].

### Assessment of GV by means of CGM

Less than 6 days of echocardiographic examination, all patients underwent CGM at least 72 h which continuously measured blood glucose level every 5 min by means of a commercially available CGM system (iPro2, Medtronic, Northridge, CA). GV was evaluated as standard deviation (SD) of blood glucose level. The patients were then divided into two groups based on the average SD of blood glucose levels (35.9 mg/dL).

### Statistical analysis

Continuous variables were expressed as mean values with SD for normally distributed data and as medians values with interquartile range for non-normally distributed data. Categorical variables were expressed as frequencies and percentages. The parameters of the two subgroups were compared by using Student t test or Mann–Whitney U test as appropriate. Proportional differences were evaluated with Fisher’s exact test. The initial univariate logistic regression analysis to identify univariate determinants of LV diastolic dysfunction (E/e’ > 14) was followed by a multivariate logistic regression model using stepwise selection, with the p levels for entry from the model set at < 0.10. Sequential logistic models were performed to determine the incremental benefit of GV in relation to clinical variables including age, gender, hypertension, and HbA1c. A statistically significant increase in the global log-likelihood χ^2^ of the model was defined as representing an incremental predictive value. For all steps, a p value of < 0.05 was considered statistically significant. All analyses were performed with commercially available software (MedCalc software version 19.0.7.; MedCalc Software, Mariakerke, Belgium).

## Results

### Comparison between clinical and echocardiographic characteristics of high and low GV groups

**Fig. 1:**
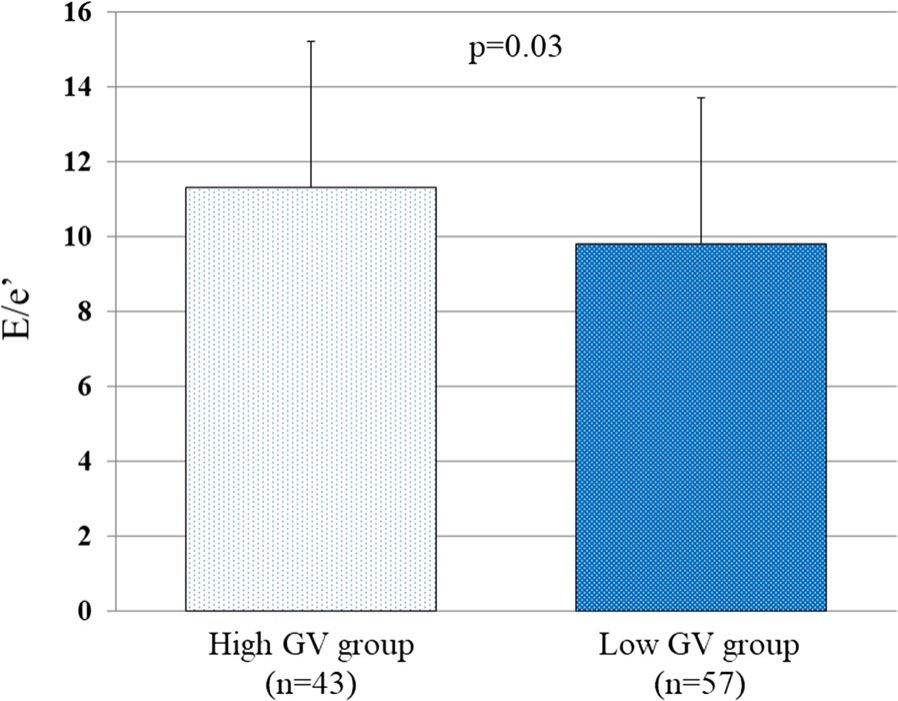
Bar graphs of E/e’ of high and low GV groups, showing significantly higher E/e’ in the high GV group

The baseline clinical and echocardiographic characteristics of the 100 T2DM patients are summarized in Table 1. Their mean age was 60 ± 14 years, LVEF was 65.6 ± 4.9%, and 45 patients (45%) were female. The high GV group comprised 43 T2DM patients (43%) with an average SD for blood glucose level of ≥ 35.9 mg/dL and the remaining 57 (57%) were classified as the low GV group. The clinical and echocardiographic characteristics of the high and low GV groups are summarized in Table 2. Most of the clinical and echocardiographic characteristics were similar, but one important difference was that E/e’ of the high GV group was significantly higher than that of the low GV group (11.3 ± 3.9 vs. 9.8 ± 2.8, p = 0.03; Fig. 1).

**Table 1:** Baseline characteristics of patients

Variables	All patients (n = 100)
Clinical characteristics	
Age, years	60 ± 14
Gender (female), n (%)	45 (45)
DM duration, years	10 (0.1–42)
Body weight, kg	67 ± 16
BSA, m^2^	1.70 ± 0.22
Systolic blood pressure, mmHg	129 ± 19
Heart rate, bpm	74 ± 10
BUN, mg/dL	15.6 ± 5.7
Creatinine, mg/dL	0.86 ± 0.43
eGFR, mL/min/1.73 m^2^	73.4 ± 25.4
HbA1c, %	8.5 ± 1.9
Comorbidities, n (%)	
Hypertension	58 (58)
Dyslipidemia	63 (63)
Anti-hypertensive drugs, n (%)	
Calcium channel blockers	38 (38)
ACE inhibitor/ARB	54 (54)
Mineralocorticoid receptor antagonist	1 (1)
Anti-diabetic drugs, n (%)	
DPP-4 inhibitor	51 (51)
GLP-1 RA	11 (11)
SU	28 (28)
α-GI	23 (23)
Thiazalidine	8 (8)
Metformin	57 (57)
SGLT2 inhibitor	3 (3)
Echocardiographic parameters	
LV end-diastolic volume, mL	76.0 ± 22.0
LV end-systolic volume, mL	26.8 ± 10.7
LVEF, %	65.6 ± 4.9
LVMI, g/m^2^	80.0 ± 19.0
LAVI, mL/m^2^	30.1 ± 8.0
e’, cm/s	6.05 ± 1.67
E/e’	10.5 ± 3.4
Trans-mitral flow	
E, cm/s	59.4 ± 13.7
DcT, msec	207 ± 53
E/A	0.8 ± 0.2
Pulmonary venous flow	
S, cm/s	65.4 ± 16.1
D, cm/s	41.3 ± 8.0
S/D	1.6 ± 0.4
A, cm/s	38.2 ± 20.0

Values are mean ± SD for normally distributed data and median and interquartile range for non-normally distributed data, or n (%). Assessment of pulmonary venous flow was available in 90 patients

*DM* diabetes mellitus, *BSA* body surface area, *BUN* blood urea nitrogen, *eGFR* estimated glomerular filtration rate, *ACE* angiotensin-converting enzyme, *ARB* angiotensin II receptor blocker, *DPP-4* Dipeptidyl Peptidase-4, *GLP-1 RA* glucagon-like peptide-1 receptors agonists, *SU* Sulfonylureas, *α-GI* α-glucosidase inhibitors, *SGLT2* Sodium glucose cotransporter type 2, *LVEF* left ventricular ejection fraction, *LVMI* left ventricular mass index, *LAVI* left atrial volume index, e’ spectral pulsed-wave Doppler-derived early diastolic velocity from the septal mitral annulus, E peak early diastolic mitral flow velocity, DcT E wave deceleration time, E/A peak early and late diastolic mitral flow velocity ratio, S peak systolic velocity of pulmonary venous flow, D peak diastolic velocity of pulmonary venous flow, A peak velocity of pulmonary venous flow during atrial systole

**Table 2:** Comparison of variables between high and low GV groups

Variables	High GV group (n = 43)	Low GV group (n = 57)	p value
Clinical characteristics			
Age, years	61.2 ± 15.0	59.4 ± 13.1	0.53
Gender (female), n (%)	19 (44)	26 (46)	0.89
DM duration, years	12 (0.5–42)	8 (0.1–34)	0.04
Body weight, kg	63.7 ± 13.4	69.6 ± 17.0	0.07
BSA, m^2^	1.67 ± 0.19	1.73 ± 0.24	0.15
Systolic blood pressure, mmHg	131 ± 21	128 ± 17	0.44
Heart rate, bpm	72 ± 10	76 ± 10	0.05
BUN, mg/dL	16.5 ± 5.8	15.0 ± 5.6	0.20
Creatinine, mg/dL	0.93 ± 0.45	0.80 ± 0.40	0.13
eGFR, mL/min/1.73 m^2^	66.2 ± 22.8	78.8 ± 25.9	0.01
HbA1c, %	8.7 ± 1.9	8.3 ± 2.0	0.23
Comorbidities, n (%)			
Hypertension	26 (60)	32 (56)	0.67
Dyslipidemia	25 (58)	38 (67)	0.39
Anti-hypertensive drugs, n (%)			
Calcium channel blockers	22 (37)	16 (39)	0.89
ACE inhibitor/ARB	32 (56)	22 (51)	0.63
Mineralocorticoid receptor antagonist	1 (2)	0 (0)	0.25
Anti-diabetic drugs, n (%)			
DPP-4 inhibitor	20 (47)	31 (54)	0.44
GLP-1 RA	3 (7)	8 (14)	0.27
SU	15 (35)	12 (23)	0.19
α-GI	10 (23)	13 (23)	0.96
Thiazalidine	2 (5)	6 (11)	0.29
Metformin	18 (42)	39 (68)	0.007
SGLT2 inhibitor	0 (0)	3 (5)	0.13
Echocardiographic parameters			
LV end-diastolic volume, mL	76.1 ± 23.4	75.9 ± 20.9	0.97
LV end-systolic volume, mL	25.9 ± 11.3	27.5 ± 10.2	0.48
LVEF, %	66.8 ± 5.4	64.8 ± 4.3	0.04
LVMI, g/m^2^	81.7 ± 19.4	78.5 ± 18.5	0.41
LAVI, mL/m^2^	31.4 ± 8.8	29.2 ± 7.1	0.17
e’, cm/s	5.9 ± 1.8	6.2 ± 1.5	0.44
E/e’	11.3 ± 3.9	9.8 ± 2.8	0.03
Trans-mitral flow			
E, cm/s	61.1 ± 12.8	58.1 ± 14.2	0.28
DcT, msec	210 ± 49	205 ± 55	0.59
E/A	0.8 ± 0.2	0.8 ± 0.3	0.75
Pulmonary venous flow			
S, cm/s	67.2 ± 16.8	64.0 ± 15.3	0.36
D, cm/s	43.3 ± 8.4	40.0 ± 7.3	0.04
S/D	1.6 ± 0.4	1.6 ± 0.7	0.65
A, cm/s	42.5 ± 24.9	34.8 ± 13.8	0.07

Values are mean ± SD for normally distributed data and median and interquartile range for non-normally distributed data, or n (%)

Assessment of pulmonary venous flow was available in 40 patients (High GV group) and 50 patients (Low GV group)

*GV* glycemic viability

All other abbreviation as in Table 1

### Association of GV with LV diastolic function

**Fig. 2:**
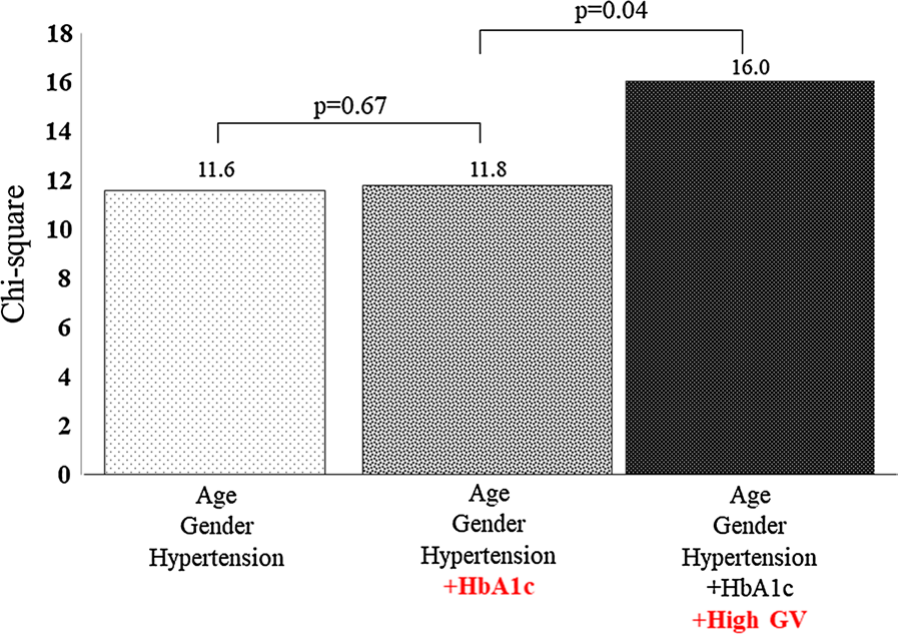
The incremental benefits determined by means of sequential logistic models of the association of LV diastolic dysfunction. The model shown here, based on clinical variables including age, gender and hypertension, disclosed no improvement for the addition of HbA1c, but did show improvement for the addition of high GV

Table 3 shows the results of the univariate and multivariate logistic regression analyses for the association of GV with LV diastolic dysfunction, defined as E/e’ > 14 for T2DM patients. An important finding of the multivariate regression analysis was that high GV, defined as an average SD for blood glucose level of ≥ 35.9 mg/dL, was an independent determinant parameter, as was age, for LV diastolic dysfunction (OR 3.670; 95% CI 1.019–13.220; p = 0.047). Furthermore, the incremental benefits determined by means of sequential logistic models of the association of LV diastolic dysfunction are shown in Fig. 2. One model, based on clinical variables including age, gender and hypertension (χ^2^ = 11.6), showed no improvement for the addition of HbA1c (χ^2^ = 11.8, p = 0.67), but did show improvement for the addition of high GV (χ^2^ = 16.0, p = 0.04).

**Table 3:** Associated factor of LV diastolic dysfunction

	Univariate			Multivariate		
	OR	95% CI	p value	OR	95% CI	p value
Age	1.077	1.017–1.141	0.011	1.070	1.012–1.131	0.017
Female	2.500	0.773–8.089	0.126			
Body surface area	0.076	0.004–1.293	0.075			
Hypertension	5.217	1.101–24.724	0.037			
Dyslipidemia	1.067	0.329–3.462	0.914			
HbA1c	1.042	0.788–1.378	0.774			
High GV	4.015	1.164–13.852	0.028	3.670	1.019–13.220	0.047
LVEF	1.176	1.018–1.385	0.774			
LVMI	1.027	0.998–1.057	0.074			
LAVI	1.06	0.9899–1.1349	0.095			

All other abbreviations as in Tables 1 and 2

*OR* odds ratio, *CI* confidential interval

**Fig. 3:**
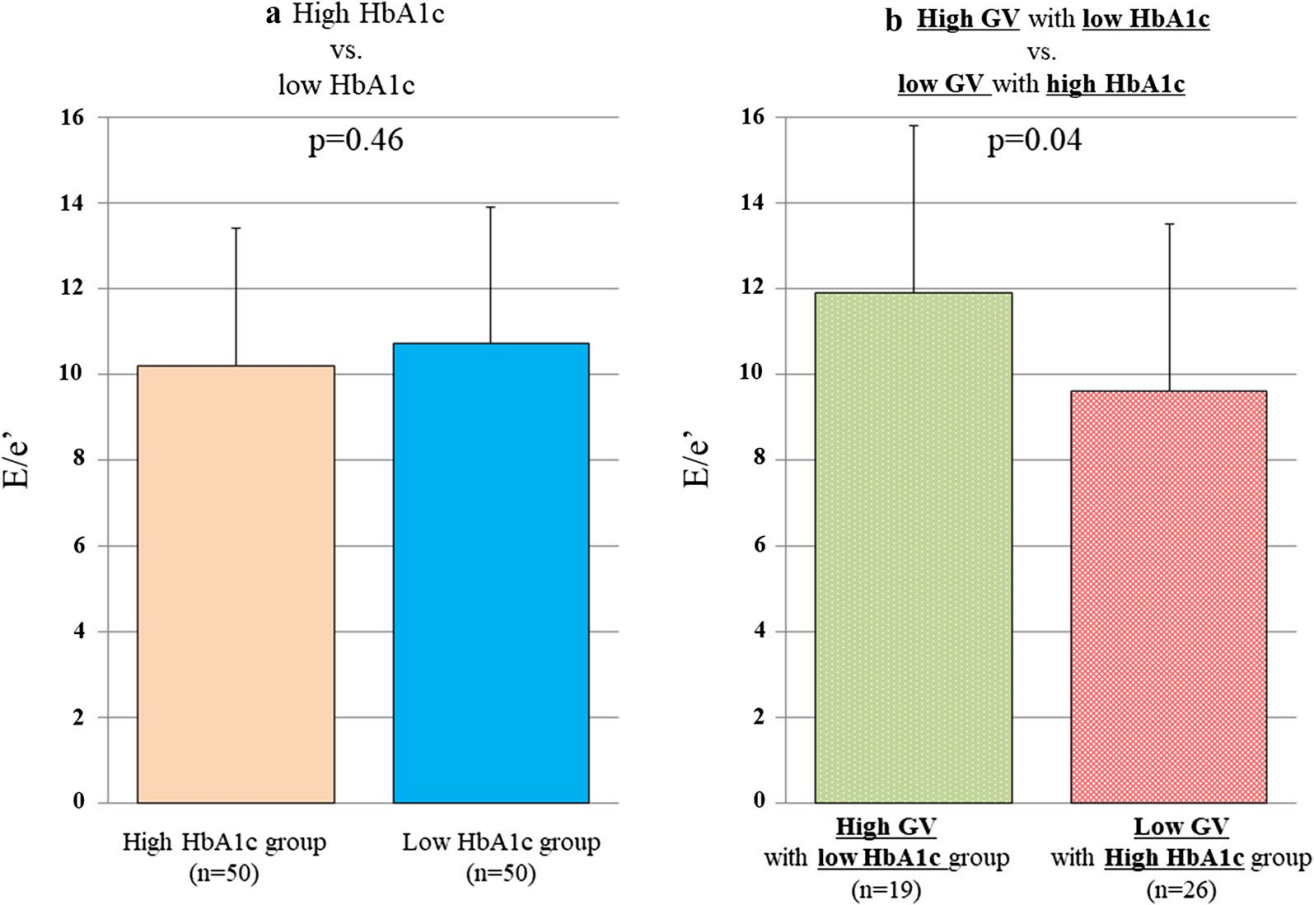
**a** Bar graphs of E/e’ for the high and low HbA1c groups, showing similar E/e’ for both groups. **b** Bar graphs of E/e’ for the high GV group with low HbA1c, and for the low GV group with high HbA1c, showing E/e’ for the high GV with low HbA1c group was significantly higher than that for patients with low GV in the high HbA1c group

Next, all patients were divided into two groups based on the median value of HbA1c (8.2 mg/dL). E/e’ for the high (≥ 8.2 mg/dL) and low (< 8.2 mg/dL) HbA1c groups was similar (10.2 ± 3.2 vs. 10.7 ± 3.5, p = 0.46; Fig. 3a), but that for patients with high GV in the low HbA1c group was significantly higher than that for patients with low GV in the high HbA1c group (11.9 ± 4.3 vs. 9.6 ± 3.0, p = 0.04; Fig. 3b).

## Discussion

The findings of our study indicate that LV diastolic function in the high GV group of asymptomatic T2DM patients with preserved LVEF was significantly worse than that in the low GV patient group. In addition, high GV was independently associated with LV diastolic dysfunction, and also added significantly to the predictive value of LV diastolic dysfunction.

### Importance of GV in T2DM

Both HF and DM strongly influence each other, with the onset of one portending a worse prognosis and further disease progression for the other. The Framingham Study reported a twofold increase in frequency of HF for men with DM and a fivefold increase for women with DM, independent of coronary artery disease and hypertension [14]. This relationship between DM and HF is the result of structural, metabolic, and functional impairment. Hyperglycemia may cause structural alterations, including microvascular remodeling and cardiac fibrosis, a finding hypothesized to be secondary to the accumulation of advanced glycation end products [15]. Thus, suboptimal glycemic control in DM and impaired insulin sensitivity have been directly correlated with an increased risk of developing HF [5, 6]. Moreover, higher HbA1c in HF patients has been associated with increased mortality [16, 17], but other studies have found a paradoxical or J-shaped relationship between HbA1c and outcomes, indicating that hypoglycemia may mitigate the possible benefits of lower HbA1c [18, 19]. CGM, on the other hand, reportedly has the potential to uncover patterns in glucose control which are not captured by HbA1c, and GV has specific clinical implications, as well as different significance from those of classical markers such as HbA1c [9], and is also strongly associated with the progression of coronary artery disease in T2DM patients [10]. Furthermore, GV was found to be independently related to carotid intima-media thickness and may contribute to the development of atherosclerosis in individuals with diabetes independent of other risk factors [20, 21]. Other findings showed GV to be a specific trigger for oxidative stress [22], which reportedly promotes inflammation and endothelial dysfunction resulting in atherosclerosis [23]. Previous research has also suggested that GV plays an important role in the development of complications related to impaired glucose metabolism.

As described previously, both HF and DM closely influence each other, with DM in particular being a major cause of HFpEF, which usually presents as LV diastolic dysfunction. However, the impact of GV on LV function, especially LV diastolic function in asymptomatic T2DM patients with preserved LVEF, remains unclear. However, LV diastolic dysfunction caused by DM-related cardiac abnormality is identifiable as the earliest functional alteration in the course of T2DM patients [24–26], while T2DM is known as a significant factor associated with HFpEF as well as hypertension or obesity. This accounts for the fact that LV diastolic dysfunction is the classical and most frequently observed early LV functional abnormality in T2DM patients [27], and asymptomatic LV diastolic dysfunction has been detected in up to 75% of normotensive T2DM patients without evident coronary artery disease [28]. For the asymptomatic T2DM patients with preserved LVEF in our study, LV diastolic function of the high GV group was significantly worse than that of the low GV group, while high GV was independently associated with LV diastolic dysfunction. Although the mechanism for the association of GV with LV diastolic function is not yet fully understood, preliminary studies suggest glycemic fluctuations play a role in promoting endothelial toxicity, oxidative stress, and ischemia [22, 29, 30]. Furthermore, it has been reported that rapid glucose swings are also associated with more profound endothelial toxicity than are tonic glucose elevations in vitro [29]. Thus, the main importance of these findings may lie in the potential application of interventions that target glycemic variability.

Moreover, it is recently reported that sitagliptin, a dipeptidyl peptidase-4 (DPP-4) inhibitor-enhanced glucagon-like peptide-1 may ameliorate LV diastolic dysfunction in T2DM by shifting fatty acid to glucose utilization in the cardiomyocyte, and thus, improving cardiac efficiency and reducing lipolysis [31–33]. Therefore, DPP-4 inhibitor might be one of the therapeutic options for the prevention of the future development of HFpEF in T2DM patients.

### Clinical implication

As already mentioned the pathogenesis of DM-related cardiac dysfunction is thought to be multifactorial, and possibly a key factor in the development of HFpEF in T2DM patients, which presents as LV diastolic dysfunction. It has also been previously reported that simple suboptimal glycemic control, such as seen in high HbA1c, was associated with the development of HF in patients with newly diagnosed DM, while a 1% reduction in HbA1c was associated with a 16% risk reduction in the development of HF in patients with newly diagnosed T2DM [34]. Since there is no effective pharmacological therapy for patients with HFpEF, GV may be another possible therapeutic target for preventing the future development of HFpEF as well as for simple glycemic control in asymptomatic T2DM patients with preserved LVEF without coronary artery disease. Interestingly, LV diastolic function even in patients with high GV and low HbA1c was found to be significantly worse than that in patients with low GV but high HbA1c. A subject for future studies can thus be to determine what kind of antihyperglycemic drugs should be used for the improvement of GV in T2DM patients.

### Study limitations

This study covered a small number of patients, so that further prospective studies with larger patient populations will be needed to validate our findings. The most common parameter of GV is the mean amplitude of glycemic excursion (MAGE), which has been determined by calculating the arithmetic mean of the difference between consecutive peaks and nadir if the difference is > 1 SD of the mean glucose level [35]. Since calculating MAGE for all patients was not possible due to this being a retrospective study, GV was evaluated as SD of blood glucose levels. Finally, only a small number of patients were available for follow-up data after therapeutic intervention for T2DM, so that the effect of therapeutic intervention for GV on LV diastolic function remains unclear.

## Conclusion

GV appears to be an important associated factor for LV diastolic function in asymptomatic T2DM patients with preserved LVEF without coronary artery disease. Since HFpEF is a complex clinical syndrome, reducing GV may represent a new therapeutic strategy for the prevention of the future development of HFpEF in such patients.

## Data Availability

Data sharing not applicable to this article as no datasets were generated or analyzed during the current study.
